# Sex- and season-dependent differences in telomere length and telomerase activity in the leaves of ash and willow

**DOI:** 10.1186/2193-1801-3-163

**Published:** 2014-03-28

**Authors:** Ying Mu, Lan-fang Ren, Zhi-li Xun, Dan-dan Zhang, Han Song, Hai Lu, Feng-lan Li, Di Liu

**Affiliations:** College of Life Sciences and Biotechnology, Beijing Forestry University, Mail-box 162#, No. 35 Qinghua East Road, Haidian District Beijing, 100083 People’s Republic of China; National Engineering Laboratory for Tree Breeding, Beijing, 100083 People’s Republic of China; Key Laboratory of Genetics and Breeding in Forest Trees and Ornamental Plants, Ministry of Education, Beijing, 100083 People’s Republic of China; The Tree and Ornamental Plant Breeding and Biotechnology Laboratory of State Forestry Administration, Beijing, 100083 People’s Republic of China

**Keywords:** Ash, Willow, Sex-specific, Season-specific, Telomere length, Telomerase activity

## Abstract

Telomeres and telomerase have important biological functions and can protect chromosome ends. In this study, sex- and season-dependent changes in telomere length and telomerase activity in ash and willow were analyzed. A statistical analysis showed that the telomere lengths of male and female trees differed significantly (P < 0.05). In ash, the telomere lengths of female trees were shorter than those of male trees. In willow, the telomere lengths of female trees were longer than those of male trees. During the annual developmental cycle, the telomere lengths of male and female ash and willow increased from April to May (P < 0.05), remained stable from May to August (P > 0.05), and decreased significantly in September and October (P < 0.05). Additionally, telomerase activities could be detected in both male and female ash and willow trees from April to October. Our results show that the telomere lengths changed according to season and sex in ash and willow. Telomere length did not have a direct positive correlation with telomerase activity.

**Electronic supplementary material:**

The online version of this article (doi:10.1186/2193-1801-3-163) contains supplementary material, which is available to authorized users.

## Background

Telomere is the end of linear eukaryotic chromosome, it is complex nucleoprotein structures consisting of repeated DNA sequences. Telomere has important biological functions, it can protect chromosome from degradation and fusion and prevent the loss of terminal sequences during DNA replication (McKnight et al. 2002). A heptanucleotide telomere repeat (TTTAGGG)_n_, is highly conserved in plants, with the exception of Asparagales and Solanaceae species (Shakirov et al. 2008). Telomere length is maintained by telomerase, which is a specialized reverse transcriptase. The basic functions of telomerase are extension of the G-overhang and replenishment of the telomere DNA sequences lost as a consequence of the end-replication problem by using its own RNA component as template (Collins 2006).

The effect of changes in telomere length and telomerase activity on the growth cycle has been explored previously. In somatic tissues of long-lived mammals, reduced telomerase activity causes shortened telomeres with each cell division, and after the telomeres reach a critical length, the cells become senescent. However, the corresponding telomerase activity of plants differs from that of animals; for example, vegetative or meristematic tissues exhibit high-level activity (McKnight et al. 2002;Vleck et al. 2003). In addition, both telomerase and alternative mechanisms have been described to control telomere length in the herbaceous plants, such as *Arabidopsis thaliana*, *Oryza sativa*, and *Zea mays* (Richards and Ausubel 1988;Heller-Uszynska et al. 2002;Li et al. 2009), and in the perennial trees *Carica papaya* (Shakirov et al. 2008), *Pinus longaeva* (Flanary and Kletetschka 2005), *Ginkgo biloba* (Liu et al. 2007;Song et al. 2010,2011), and *Pinus sylvestris* (Aronen and Ryynänen 2012).

For long-lived trees, telomere dynamics and their connection to plant sex and the annual developmental cycle is an interesting topic that requires further study. During the annual developmental cycle, the leaf telomere lengths of *G. biloba* remained stable from April to August, but decreased significantly in September and October (Song et al. 2010). The highest level of telomerase activity was found in April, then decreased from April to October (Song et al. 2011). The telomere length in leaf tissue differed between female and male mature trees, although the difference was not statistically significant (Liu et al. 2007). However, human telomere length differences were investigated in different sexes, and it was found that males had shorter telomeres and higher attrition rates (Mayer et al. 2006). We wanted to explore whether this difference is universal in plants or found only in *G. biloba*. Until now, few studies regarding sex- and season-dependent differences in telomere length and telomerase activity in angiosperms have been performed.

Ash (*Fraxinus pennsylvanica* Mars. var. *subintegerrima* [Vahl.] Fern.) and willow (*Salix matsudana* Koidz.) are deciduous broad-leaved woody angiosperm trees that are widely distributed across China and other countries. In this study, ash and willow were used to study sex- and season-specific changes in telomere length and telomerase activity in angiosperm perennial trees.

## Results

### Significant differences in telomere length and insignificant differences in telomerase activity between male and female ash and willow trees

**Figure 1:**
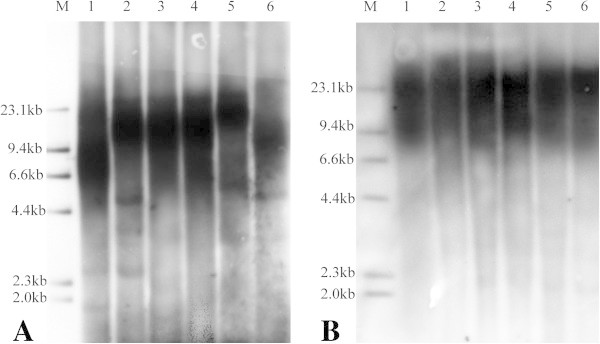
**Representative Southern hybridization analysis images used to measure the telomere length in the leaves of male or female ash and willow trees. A** Ash samples. **B** Willow samples. Lane M: DNA molecular weight marker II, digoxigenin-labeled (Roche); lanes 1–3: male trees; lanes 4–6: female trees. Leaves of ash and willow were sampled in October, 2011.

**Figure 2:**
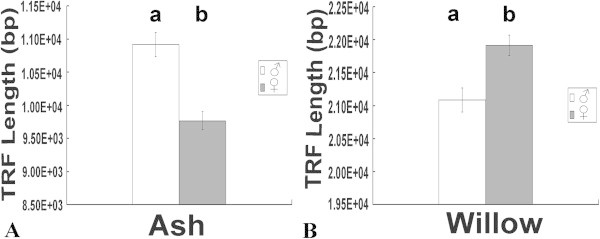
**Leaf telomere lengths in male and female ash and willow trees in April. A** Ash samples. The telomeres were longer in males than in females (P < 0.05). **B** Willow samples. The telomeres were shorter in males than in females (P < 0.05). The different lowercases above the bars indicate significance at the 0.05 level.

To measure differences in telomere length and telomerase activity between male and female ash and willow trees, leaves from ash and willow were obtained from five male trees and five female trees from April to October of 2011. Representative Southern hybridization images (the collection time of the samples was October, 2011) used for the measurement of telomere length are shown in Figure 1. Significant differences between the telomere lengths of male and female ash and willow trees were found in April (P < 0.05) (Figure 2). And samples from May to October were also analyzed to prove this difference (Additional file 1: Figure S1).

In general, significant differences between the telomere lengths of male and female ash and willow trees were found from April to September (P < 0.05). In October, the telomere lengths of male and female trees showed no significant differences for both ash and willow (P > 0.05) (Additional file 1: Figure S1). However, from April to September, the telomere lengths in male and female trees differed between ash and willow. In ash, the telomere lengths of female trees were shorter than those of male trees. In willow, the telomere lengths of female trees were longer than those of male trees.

From April to September, the telomere lengths of male ash trees were longer than those of female trees by 11.8, 10.7, 10.1, 5.1, 9.8, and 5.5% (P < 0.05), respectively. However, the telomere lengths of male willow trees were shorter than those of female trees by 4.4, 4.1, 4.0, 3.9, 4.5, and 1.6% from April to September (P < 0.05) (Additional file 1: Figure S1), respectively.

**Figure 3:**
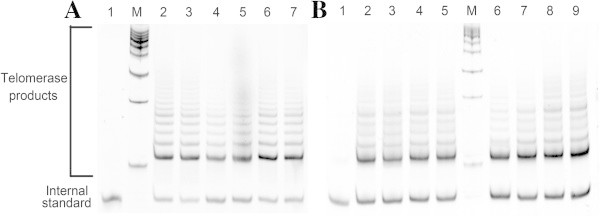
**Representative TRAP analysis images used to measure the telomerase activity in leaves of male or female ash and willow. A** Ash samples. Lane 1: telomerase-negative control M: 50-bp ladder DNA marker; lanes 2–4: male trees; lanes 5–7: female trees. **B** Willow samples. Lane 1: telomerase-negative control; lane M: 50-bp ladder DNA marker; lanes 2–5: male trees; lanes 6–9: female trees. Leaves of ash and willow were sampled in April, 2011.

**Figure 4:**
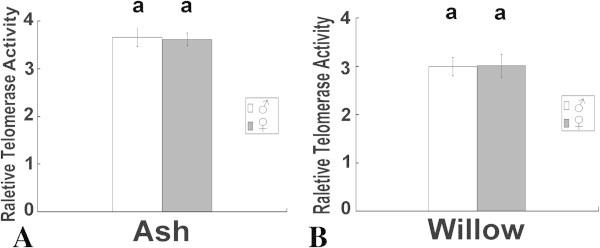
**Leaf telomerase activity level in male and female ash and willow trees in April. A** Ash samples. The telomerase activity levels of male and female trees were equal (P > 0.05). **B** Willow samples. The telomerase activity levels of male and female trees were equal (P > 0.05). The lowercases above the bars indicate significance at the 0.05 level.

To examine sex differences in leaves from ash and willow, TRAP assays were used to analyze telomerase activity. Representative TRAP analysis images used to measure the telomerase activity in leaves of male or female ash and willow. (Figure 3, the collection time of the samples was April, 2011). Our results indicate that telomerase activities could be detected in both male and female ash and willow trees from April to October (Figure 4 and Additional file 1: Figure S2).

### Season-specific changes in telomere length and telomerase activity in ash and willow

**Figure 5:**
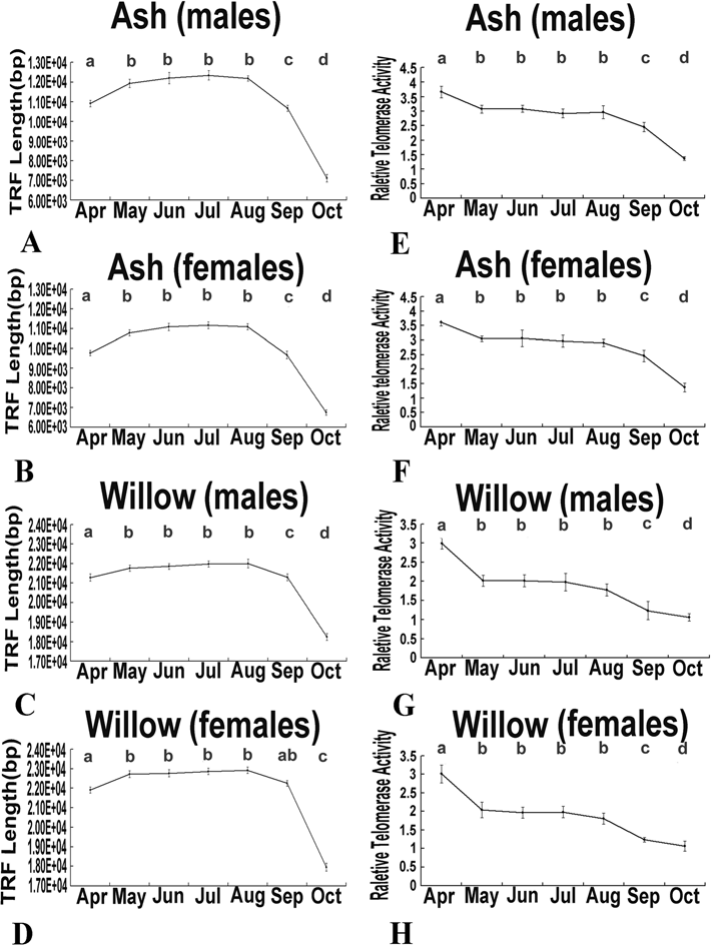
**Leaf telomere length and telomerase activity in ash and willow trees from April to October.** Leaf telomere length from April to October **(A-D)**. **A** Male ash samples. **B** Female ash samples. **C** Male willow samples. **D** Female willow samples. Leaf telomerase activity from April to October **(E-H)**. **E** Male ash samples. **F** Female ash samples. **G** Male willow samples. **H** Female willow samples. The different lowercases above the bars indicate significance at the 0.05 level.

To examine seasonal developmental changes in telomere length and telomerase activity, leaf samples were collected every month from April to October in 2011. The telomere lengths increased from April to May (P < 0.05), remained stable from May to August (P > 0.05), and decreased sharply in September and October (P < 0.05) in both male and female ash and willow trees (Figure 5A-D). From April to October, the range of telomere lengths in male and female willow trees was15 kb -24 kb and in male and female ash trees was 6 kb–14 kb (Figure 5). The telomere lengths of ash male, ash female, willow male, and willow female were 10920 ± 170 bp, 9760 ± 140 bp, 21290 ± 180 bp, 21910 ± 150 bp in April, respectively. For the ash male, ash female, willow male, and willow female groups, the telomere lengths increased by 9.3, 10.5, 3.7, and 3.6% from April to May, decreased by 12.5, 12.8, 3.4, and 2.9% from August to September, and decreased by 33.3, 30.3, 3.3, and 7.5% from September to October, respectively.

The telomerase activity levels decreased during the annual developmental cycle in both male and female ash and willow trees (Figure 5E-H). In male and female ash trees, leaf telomerase activity decreased from April to May, remained stable from May to August, and was very low in September and October (Figure 5E and F). In willow trees, the highest telomerase activity levels were observed in April, remained stable from May to August, and continued to decrease from September to October (Figure 5G and H). The telomerase activities of ash and willow were 3.60 ± 0.19 and 3.01 ± 0.24 in April, respectively. For the ash and willow trees, telomerase activity decreased by 16.1 and 32.2% from April to May, continued to decrease by 17.5 and 37.8% from August to September, and further decreased by 44.1 and 13.8% from September to October, respectively.

## Discussion

### Difference in telomere length and telomerase activity between male and female ash and willow trees

According to our results, the telomere lengths of male and female trees showed significant differences in ash and willow. However, in a previous report, statistical analyses showed that the telomere lengths of female and male trees were equal in all tested groups of ginkgo (Song et al. 2010). Previous studies showed that males and females did not have different telomere lengths in *Melandrium album* (Riha et al. 1998). On the other hand, it was found that women have longer telomeres and fewer repeat losses per year than males, and that every chromosome arm has an individual age-specific telomere length, resulting in unexpected heterogeneity in chromosome-specific regression lines (Mayer et al. 2006). Apart from the above-mentioned sex-specific discrepancies, chromosome arm-specific telomere lengths were strikingly similar in men and women. This finding is suggestive of a mechanism that specifically regulates telomere length, regardless of sex, leading to inter-chromosomal telomere variations (Mayer et al. 2006).

The reasons for the differences between dynamic changes in telomere length in males and females may include differences in sex-specific telomere restructuring and recombination (Pfeifer et al. 2003), selective mortality, cell populations, and telomerase expression (Bischoff et al. 2005). The results in ash may be explained by the fact that the leaves of male trees grow earlier and faster than those of females in April, while from May to September, there is no obvious morphological difference between male and female, and the difference of telomere length between male and female ash trees were consistent from April to September, phenology may be another reason, but the results more related to sex in the annual developmental cycle. However, there is no obvious morphological difference between the leaves of male and female willows in April. The reason for this phenomenon in willow trees may be the initial telomere length of male trees, which is shorter than that of females. In addition, the difference between species may be another important factor in the different telomere lengths of male and female trees. The influence of sex differentiation on telomere length in animals has been studied, but the mechanisms require further investigation.

Generally, male trees are used as street and garden trees because of their influence on the environment, resistance, and survival rate. However, early sex identification is difficult prior to the first flowering. The difference in telomere length between male and female trees may be used to address this problem.

### Correlation between telomere length and telomerase activity in ash and willow during the annual developmental cycle

The telomere lengths and telomerase activity levels of ash and willow trees changed seasonally and showed a regular trend. In this study, the telomere length did not peak with telomerase activity in April. From May to August, the telomere lengths peaked and remained stable during the annual developmental cycle, whereas the telomerase activity level decreased and remained stable in ash and willow trees. In September and October, the telomere length and telomerase activity level decreased significantly (Figure 5). Thus, the telomere length and telomerase activity level in ash and willow trees during the annual developmental cycle were correlated. The model of telomere length homeostasis for the Columbia ecotype of *Arabidopsis* reported by Shakirov and Shippen (2004) may be able to explain the phenomenon observed in our study. They hypothesized that telomeres reach an optimal, ecotype-specific size based on the opposing consequences of telomerase activity and the end-replication problem. Telomerase is more likely to function when the telomere length reaches a lower threshold. As telomeres approach the optimal threshold, there is an equal chance of telomerase action or inaction, resulting in telomere splitting (middle telomere). At the maximum telomere size, telomerase is less likely to function and telomeres shorten because of the end-replication problem (Shakirov and Shippen 2004).

In April, telomerase activity peaked when the telomere lengths of ash and willow trees reached a critical value. However, the critical telomere length value may not be the minimum. In spring, most trees began to sprout and cell division was very rapid. Therefore, as the most important enzyme of the chromosome end-repair mechanism, the telomerase activity level is inevitably high. However, at the same time, chromosome ends are constantly damaged because of the rapid division of leaf cells. Thus, the telomere length is small. From May to August, the growth situation of the plant is more stable; therefore, the telomere length and telomerase activity level are stable during this time. However, in September and October, although the telomere lengths were very small, telomerase activity was also low. These findings may reflect leaf cell apoptosis. A study of genome degradation in tobacco BY-2 cells showed that tobacco cells exposed to cold stress undergo specific apoptotic changes, including the non-random degradation of nuclear chromatin (Koukalova et al. 1997). In September and October in Beijing, the monthly mean temperatures are below 15°C, while from May to August the monthly mean temperatures are above 25°C. Moreover, ash and willow are both deciduous broad-leaf tree species. From the middle of September, these trees begin to shed their leaves. Therefore, the decrease in telomere length may arise from an early-stage apoptotic process. Telomerase activity decreases at the same time. Thus, telomere length does not have a simple positive correlation with telomerase activity. Optimal telomere length is regulated and maintained by telomerase activity. Increasing or decreasing telomerase activity hysteresis depends on telomere length. Both telomere length and telomerase activity may be under the influence of apoptosis.

## Materials and methods

### Sample collection

Considering the reliability and feasibility, leaves were chosen as experimental materials. Leaves from ash and willow trees of the same age (about 50 and 35 years old, respectively) were obtained from free-grown trees (five male and five female trees) on the campus of Beijing Forestry University (Beijing, China). The leaves which had been sampled were the first leaves formed in that growing season, and the leaves were sampled in the same way in all the trees.

Leaves of both ash and willow were collected from the current-year branch which growing at the bottom of the trees and sampled every month from the 19th to the 22nd from April to October of 2011. Each sample was fixed in liquid nitrogen and stored at −80°C.

### Determination of telomere length

TRFL, as an indicator of telomere length, was determined by Southern hybridization analysis (Nakamura et al. 2002). Samples were placed in a mortar and ground to a fine powder using a pestle and liquid nitrogen. Genomic DNA was prepared from each sample using the hot CTAB method and quantified by mrcio-spectrophotography (NANODROP 2000, Thermo). The integrity of the DNA samples was confirmed by ethidium bromide staining of the gel before blotting. Aliquots of DNA (approximately 20 μg) were digested for 36 h with restriction enzymes (ash with *Hin*fI and willow with *Eco*RI), and the digestion products were loaded onto a horizontal 0.8% agarose gel (6.5 × 10 cm) and electrophoresed in 1× TAE buffer for approximately 3 h at 100 V at room temperature. Southern hybridization was performed as described previously (Liu et al. 2007;Song et al. 2010) using a DIG High Prime DNA Labeling and Detection Starter Kit II (Roche, Basel, Switzerland) with an end digoxigenin-labeled complementary telomere-specific oligonucleotide probe (5′-CCCTAAACCCTAAACCCTAAACCC-3′). The telomere lengths were measured as described previously (Liu et al. 2007;Song et al. 2010).

### Determination of telomerase activity

Telomerase activity was analyzed using the telomeric repeat amplification protocol (TRAP) (Kim and Wu 1997;Flanary and Streit 2003;Song et al. 2011). There are four basic steps in TRAP method: 1) Extract telomerase: in this step, CHAPS lysis buffer (0.5% CHAPS) was used to extract telomerase. And a Coomassie protein determination method (BSA Reagent was the standard) was used to determine the protein concentration of the telomerase extract. 2) Telomerase extension: Each 50 μl reaction initially contained 5 μl 10 × TRAP buffer, 200 μM dNTP, 100 ng TS primer (5′AATCCGTCGAGCAGAGTT-3′), 500 ng telomerase extract, and RNAase-free water up to 50 μl. This mixture was incubated for 30 min at room temperature to allow telomerase to add telomeric repeats. 3) RCR amplification: PCR was performed as follows, initial denaturation at 94°C for 2 min, followed by 30 cycles of 94°C for 30 s, 60°C for 30 s, and 72°C for 30 s. CHAPS buffer was used as a negative control and an internal standard TSNT (5′-AATCCGTCGAGCAGAGTTAAAAGGCCGAGAAGCGAT-3′) was used in this step. 4) Electrophoresis and measurement: after 12.5% non-denaturing polyacrylamide gel electrophoresis, the telomerase products were visualized by staining with ethidium bromide and photographed under ultraviolet light using an electronic gel documentation system. Quantitation of telomerase activity was performed by densitometry (Flanary and Streit 2003;Song et al. 2011).

### Statistical analysis

The measurement of telomere length for each sample was repeated three times by Southern hybridization. All telomere lengths are reported as the mean ± standard deviation. The measurement of telomerase activity for each sample was repeated three times by TRAP assay. Each telomerase activity level is reported as the mean ± standard deviation. A statistical analysis was performed by one-way analysis of variance using Matlab7.0 software (The MathWorks, Inc., Natick, MA). Results were considered statistically significant at P < 0.05. Different letters above the bars in the figures indicate significance at the 0.05 level.

## Electronic supplementary material

Additional file 1: Figure S1: Leaf TRF lengths of males and females. a ash samples. From April to September, males’ TRF lengths are longer than the females (P < 0.05). b willow samples. From April to September, willow males’ TRF lengths are shorter than the females (P < 0.05). In October, telomere lengths of male and female trees were equal for both ash and willow (P > 0.05). The different capital letters above the bars indicate significance at the 0.05 level for the same month. **Figure S2**. Leaf telomerase activity of males and females. a ash samples. telomerase activity of males and females are equal in every month (P > 0.05). b willow samples. telomerase activity of males and females are equal in every month (P > 0.05). The different capital letters above the bars indicate significance at the 0.05 level for the same month. (DOC 804 KB)

## Abbreviations

CTAB: Cetyl trimethy ammonium bromide
CHAPS: 3-[(3-Cholamidopropyl)dimethylammonio]-1-Propanesulfonate
TRAP: Telomeric repeat amplication protocol
TRFL: Telomere restriction fragment length.
