# Supplementary material for: Sex- and season-dependent differences in telomere length and telomerase activity in the leaves of ash and willow
Source: Springerplus. 2014 Mar 28;3:163. doi: 10.1186/2193-1801-3-163 (PMC3977023; doi:10.1186/2193-1801-3-163)
Supplement: Supplementary file 1 — Additional file 1: Figure S1: Leaf TRF lengths of males and females. a ash samples. From April to September, males’ TRF lengths are longer than the females (P < 0.05). b willow samples. From April to September, willow males’ TRF lengths are shorter than the females (P < 0.05). In October, telomere lengths of male and female trees were equal for both ash and willow (P > 0.05). The different capital letters above the bars indicate significance at the 0.05 level for the same month. Figure S2. Leaf telomerase activity of males and females. a ash samples. telomerase activity of males and females are equal in every month (P > 0.05). b willow samples. telomerase activity of males and females are equal in every month (P > 0.05). The different capital letters above the bars indicate significance at the 0.05 level for the same month. (DOC 804 KB) [file 40064_2014_873_MOESM1_ESM.doc]

**Additional file 1:**

**
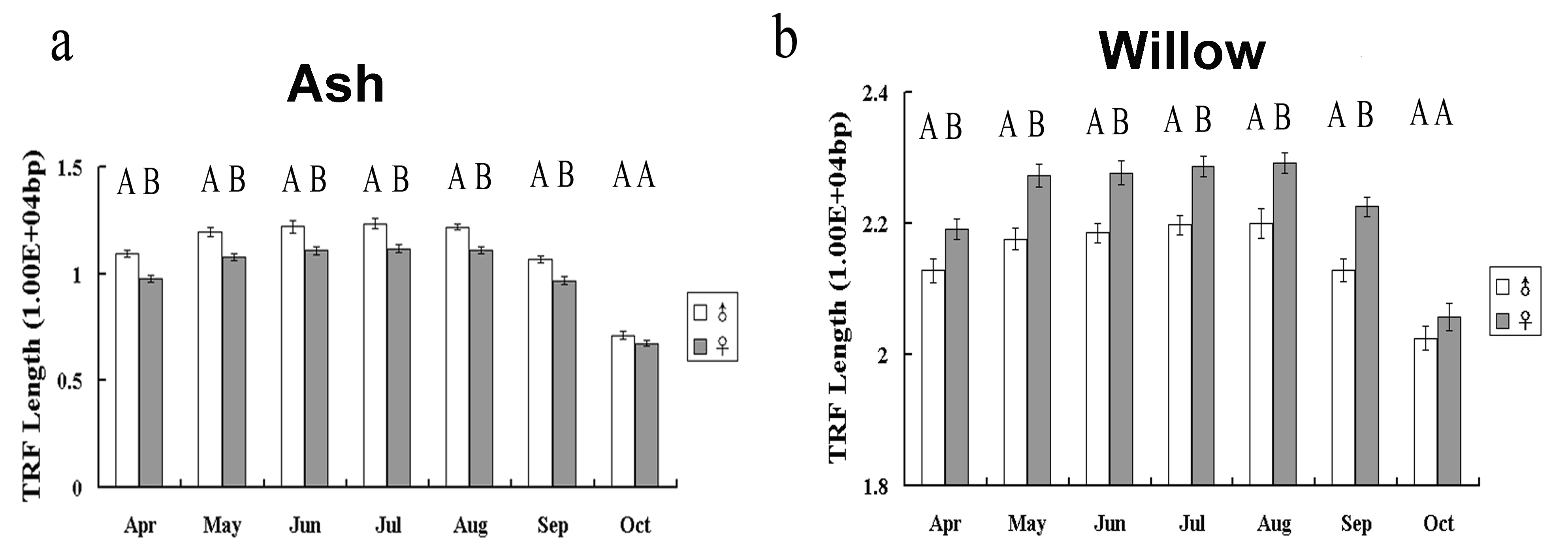
**

**Figure S1** Leaf TRF lengths of males and females. **a** ash samples. From April to September, males’ TRF lengths are longer than the females (P<0.05). **b** willow samples. From April to September, willow males’ TRF lengths are shorter than the females (P<0.05). In October, telomere lengths of male and female trees were equal for both ash and willow (P>0.05). The different capital letters above the bars indicate significance at the 0.05 level for the same month.


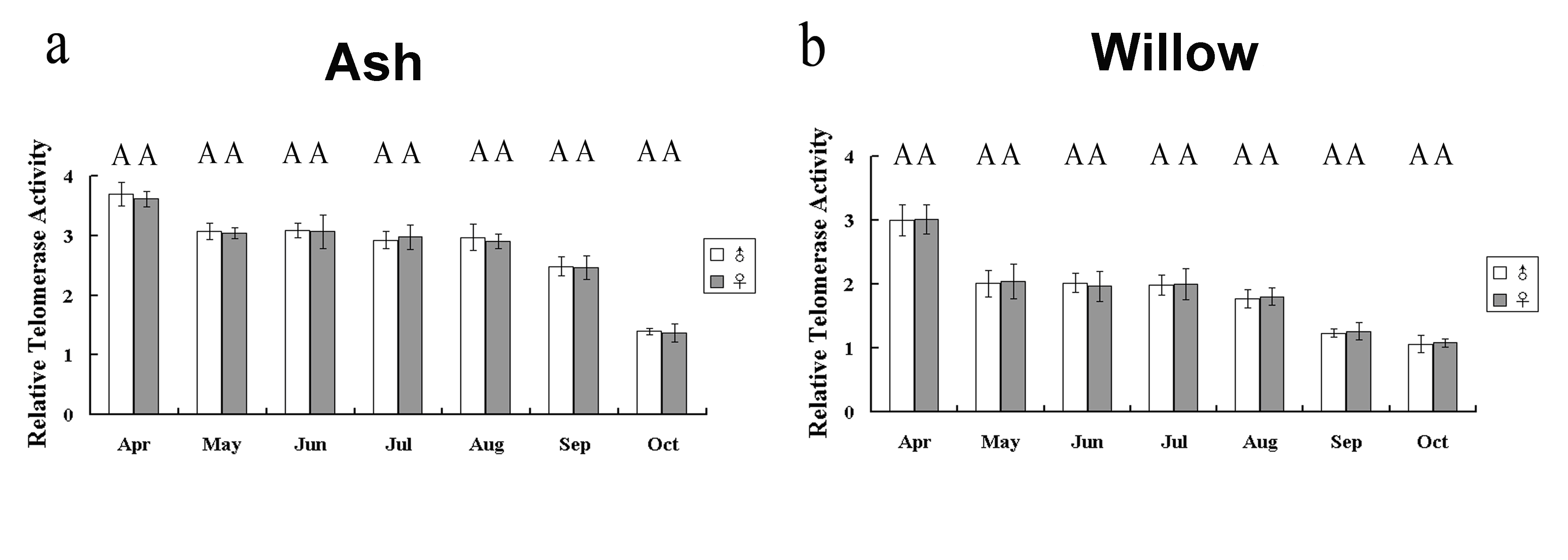


**Figure S2** Leaf telomerase activity of males and females. **a** ash samples. telomerase activity of males and females are equal in every month (P>0.05). **b** willow samples. telomerase activity of males and females are equal in every month (P>0.05). The different capital letters above the bars indicate significance at the 0.05 level for the same month.
